# The Relationship between Free Press and Under-Reporting of Non-Fatal Occupational Injuries with Data from Representative National Indicators, 2015: Focusing on the Lethality Rate of Occupational Injuries among 39 Countries

**DOI:** 10.3390/ijerph15122856

**Published:** 2018-12-14

**Authors:** Sung-Shil Lim, Jin-Ha Yoon, Jeongbae Rhie, Suk Won Bae, Jihyun Kim, Jong-Uk Won

**Affiliations:** 1 The Institute for Occupational Health, Yonsei University College of Medicine, Seoul 03722, Korea; lssmail@daum.net (S.-S.L.); flyinyou@gmail.com (J.-H.Y.); newzzanggu@hanmail.net (S.W.B.); jihyunkim0924@gmail.com (J.K.); 2 Graduate School of Public Health, Yonsei University, Seoul 03722, Korea; 3 Department of Preventive Medicine and Public Health, Yonsei University College of Medicine, Seoul 03722, Korea; 4 Department of Occupational and Environmental Medicine, Dankook University College of Medicine, Cheonan 31116, Korea; rhie76@gmail.com; 5 Department of Public Health, Yonsei University, Seoul 03722, Korea

**Keywords:** occupational injury, ILOSTAT, freedom of the press, national indicator, under-report

## Abstract

The epidemiology of occupational injuries is reported worldwide, but suspicions of under-reporting prevail, probably associated with free press. We examined the association between freedom of the press and lethality rate of occupational injuries based on the most comprehensive International Labour Organization database on labour statistics (ILOSTAT) among 39 countries. The occupational injury indices, national indicators, and information on freedom of the press in 2015 were sourced from ILOSTAT, World Bank open data, World Health Organization and Freedom House. The lethality rate was the number of fatal occupational injuries per 10,000 total occupational injuries. The relationship among fatal and total occupation injury rates, lethality rate, and national statistics were analysed using Spearman’s rank correlation coefficients. Multivariable linear regression models with bootstrap estimation to manage non-normality determined freedom of the press associated with lethality rate. Freedom of the press was significantly correlated with fatal and total occupational injury rate and lethality rate of occupational injuries. Adjusting for national indicators, only freedom of the press was associated with lethality rate per 10,000 occupational injuries in the report of ILOSTAT. The lethality rate of occupational injury reported by each country might not reflect the actual lethality, but under-reported nonfatal occupational injuries, probably relating to freedom of the press.

## 1. Introduction

Recently, government authorities and employers have become increasingly interested in industrial accidents owing to the tremendous cost of occupational injuries. It is estimated that occupational factors are responsible for 8.8% of the global burden of death because of unintentional occupational injuries worldwide [1]. In the United States, in the year 2007, the number of fatal and nonfatal occupational injuries were estimated at nearly 5600 and 8,559,000 at a cost of 660 billion and 186 billion US dollars, respectively [2]. In South Korea, although occupational injury rates have decreased from 0.77 per 100 workers in 2001 to 0.50 per 100 workers in 2015, occupational injury rates are still sizable [3,4].

Providing adequate compensation and social support to workers who have been victims of occupational accidents and their families is important in protecting their families’ basic rights [5]. However, under-reporting of occupational injuries could occur due to workers’ fear of job loss, employers’ lack of understanding of the regulations of recordkeeping and employer programs that discourage reporting to the authorities [6,7]. Reporting occupational injuries to government authorities without delay and exception can prevent economic and social difficulties for victims of industrial accident when they are involved in such accidents.

The International Labour Organization Database (ILOSTAT) is a representative labour-specific statistic used to compare labour force, employment, working time, social protection, and safety and health at work across countries based on data reported from all over the world. ILOSTAT publishes comprehensive statistics on safety and health at work including occupational injuries and status of fatal and nonfatal occupational injuries by gender and occupation worldwide every year [8]. It is important for governmental authorities to obtain the exact epidemiology of industrial accidents to establish policies for managing industrial accidents and comparing the situation of occupation al injuries among various countries. It is also important to compare their level of industrial accidents with that of other countries to know the current status of their countries. However, the statistical figures of occupational injuries officially reported by each country might not reflect the actual situations in the country [7,9]. In the United States, many occupational injuries in workplace are omitted from employers’ recordkeeping logs, resulting in under-reporting to the Bureau of Labor Statistics (BLS) [7]. For example, Rosenman et al. revealed that the current national surveillance system did not capture 61% of the work-related injuries and illnesses that occurred annually in Michigan [10].

Several harmful factors and administrative procedures have had an impact on published occupational accident rates [9]. Harmful factors are those directly affecting the incidence of occupational accidents such as physical, mental or biological exposure of workers to hazardous items or an unfavourable working environment. In a study using population-wide national survey data in Germany in 2010, higher odds of occupational injuries are correlated with the factors of male gender (odds ratio (OR) 3.16), heavy carrying (OR 2.10), awkward postures (OR 1.68) and environmental stress (OR 2.16), as well as the occupations of agricultural workers (OR 5.40) and technicians (OR 3.41), compared with occupations like clerical work or the professions [11]. However, administrative procedures, e.g., reporting systems, occupational injury compensation systems, occupational health policies and coverage of workers’ compensation insurance (e.g., commuting accidents) can distort the rate of actual industrial accidents.

The lethality rate of occupational injuries, calculated as the number of fatal cases divided by the total number of occupational injuries, is a measure of the severity of occupational injuries [9,12]. There are several factors relating to lethality of occupational injuries. In Turkey, fatality per 1000 occupational injuries was higher in male workers (25.8) than in female workers (7.7), in older workers (≥60 years old: 242.1) than in younger ones (15–29 years old: 7.6) and in construction workers (73.4) than in wholesale and retail trade workers (29.0) [12]. Among the Organisation for Economic Co-operation and Development (OECD) countries, lethality rate was associated with artificial factors associated with administrative procedures such as the waiting period before workers’ compensation insurance coverage rather than actual severity of occupational injuries [9]. However, these studies focused on the status of OECD countries and did not reveal the relationship between lethality of occupational injuries and freedom of the press, which might be the cause of the under-reporting.

Freedom of expression is a facet of freedom of speech, which is declared in the Universal Declaration of Human Rights [13]. Freedom of the press refers to freedom of communication and expression through any means of transmission including electronic media and publications. The media act as a bridgehead for providing information about national and social problems, making them part of the social agenda, which often leads to policy creation to address these problems [14]. A free press could reduce corruption, induce economic development and become a bridge between government and citizens [15,16,17]. Therefore, a free press might be associated with an environment conducive to reporting to government authorities and more accurate national statistics on occupational injuries.

Therefore, our study aimed to determine whether the occupational injury indices, especially the lethality rate of occupational injuries, reported by 39 countries reflected the actual situation of accidents or under-reporting. Furthermore, it examined the relationship between national statistics, especially, freedom of the press, and the lethality rate of occupational injuries based on the statistics of ILOSTAT, World Bank open data, the World Health Organization (WHO) and Freedom House in the year 2015.

## 2. Materials and Methods

### 2.1. Occupational Injury Indices

In this study, statistics on occupational injury in 2015 were obtained for ‘fatal occupational injuries per 100,000 workers by sex and migrant status’ and ‘nonfatal occupational injuries per 100,000 workers by sex and migrant status’ in the section of safety and health at work in ILOSTAT [18] with matching data source of two occupational injury indices. Total occupational injury was calculated as the sum of fatal and nonfatal occupational injuries per 100,000 workers. The data of 43 countries from ILOSTAT had both occupational injury indices in matched data. The statistics of occupational injuries in South Korea were based on the data of the 2015 Korea Occupational Safety and Health Agency (KOSHA) Annual Report [4] due to lack of information about nonfatal occupational injuries in South Korea in ILOSTAT. Due to missing values for other national indicators in five countries, we finally studied 39 countries in total. The occupational indices were sourced from insurance records (Austria, Belgium, Bulgaria, Costa Rica, Germany, Spain, France, United Kingdom, Greece, Croatia, Italy, Republic of Korea, Luxembourg, Mexico, Portugal and Turkey); establishment surveys (Kazakhstan: businesses and organizations register; Belarus: labour-related establishment survey; Poland: statistical card on accident at work; Russian Federation: establishments sample survey on employees’ wages by occupation; Ukraine: report on occupational injuries); labour inspectorate records (Cyprus, Estonia, Hungary, Ireland, Kyrgyzstan, Lithuania, Latvia, Norway, Romania, Singapore and Slovakia); and from other administrative records and related sources (Colombia, Denmark, Finland, Malta, Malaysia, Slovenia and Sweden). We defined the lethality rate as the number of fatal occupational injuries per 10,000 total occupational injuries. Data sources were dichotomised into insurance record and others (others = 0, insurance records = 1).

### 2.2. National Indicators

Gross domestic product (GDP) per capita (constant 2011 international $) represents GDP divided by mid-year population. GDP per capita is considered an indicator of the standard of living of a country [19]. Current health expenditure (CHE) as percentage of GDP (%) is the level of current health expenditures including healthcare goods and services consumed during each year expressed as a percentage of GDP [20].

Job-related indicators include proportion of employment at skill level 1, ratio of female to male labour-force participation rate, and share of industry in total employment (%) [21]. ILO estimates of employment divided occupation into skill level 1 (low): elementary occupations; skill level 2 (medium): clerical, service and sales workers; skilled agricultural and trades workers; plant and machine operators; and assemblers; and skill levels 3 and 4 (high): managers, professionals and technicians. We defined the variable of ‘skill level 1’ as the proportion of skill level 1 to total employment, of which workers are considered vulnerable to occupational injuries. Share of industry in total employment (%) is the proportion of industry comprising mining and quarrying, manufacturing, construction, and public utilities (electricity, gas and water), in accordance with divisions 2–5 (ISIC 2) or categories C–F (ISIC 3) or categories B–F (ISIC 4) expressed as a percentage of total employment [22].

The 2016 edition of the report Freedom of the Press, which provides analytical reports and numerical scores for 199 countries and territories, constitutes a process conducted by Freedom House, an independent watchdog organization based in the United States, since 1980 [23]. Each country and territory is given a total press freedom score from 0 (best) to 100 (worst) based on 23 methodology questions consisting of three subcategories: legal environment, political environment, and the economic environment of the press. The score in terms of the political environment is weighted most heavily (legal environment: 40/100; political environment: 30/100; and economic environment: 30/100). The freedom of the press of each country was assessed by more than 90 analysts using information from field research, professional contacts, reports from local and international nongovernmental organizations (NGOs), reports of governments and multilateral bodies, and domestic and international news media. The 2016 edition of Freedom of the Press covered the status of press freedom between 1 January 2015, and 31 December 2015. Martin et al. provides a detailed description and validity of the score of the freedom of the press in Freedom House [24].

### 2.3. Data Analysis

Descriptive statistics are expressed as means and standard deviations, medians and interquartile ranges (IQR) where appropriate to characterize national indicators. The relationship between fatal, total occupation injury rate, lethality rate and national statistics was analysed using Spearman’s rank correlation coefficients due to the small number of countries, resulting in unexpected normal distribution. Three occupational injury indices were not normally distributed (Shapiro–Wilk test: all *p*-values < 0.005). The bootstrap is a simulation technique by which repeated samples are drawn with replacement from the data set when parametric assumptions are invalid [25]. Multivariable linear regression models with bootstrap estimation (the number of bootstrap replicates = 10,000) to manage non-normality were used to determine national statistics associated with lethality rate after adjusting for national indicators (data source, GDP per capita, CHE as percentage of GDP, freedom of the press, proportion of employment of skill level 1, ratio of female to male labour force participation rate and share of industry in total employment) [26]. Confidence intervals were constructed by a bootstrapping regression based on the observations and errors resampling approaches. We calculated confidence interval using the 95% bootstrap percentile interval and bias-corrected accelerated (BCa) interval. A detailed methodology of the analytical method is described in detail in this paper [27,28]. To show the trend between the factors in figures, a widely used smoothing procedure was performed with the loess smoothing algorithm (loess span = 0.75) [29]. All analyses were conducted using R (R Foundation for Statistical Computing, Vienna, Austria).

## 3. Results

### 3.1. The Basic Characteristics of Occupational Injuries and Summary Statistics among 39 Countries

Table 1 shows the descriptive characteristics of country estimates. The median of fatal and total occupational injuries per 100,000 workers among 39 countries was 2.6 and 744.9, respectively. The median of lethality rates per 10,000 occupational injuries was 29.5.

**Figure 1:**
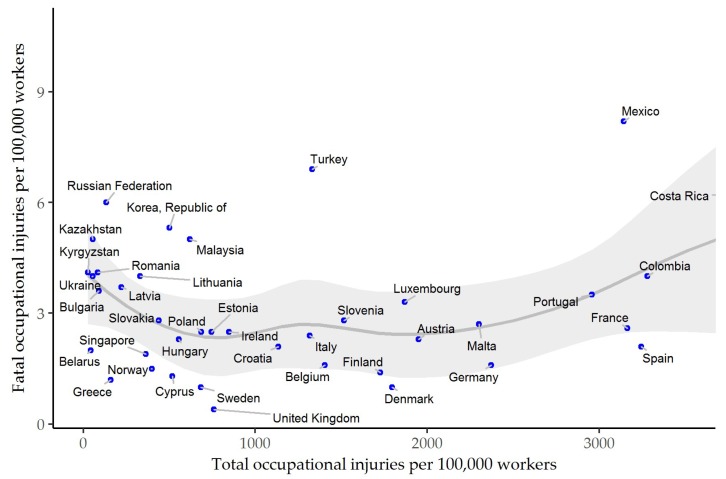
The number of total and fatal occupational injuries per 100,000 workers of each country from the data of ILOSTAT in 2015. Note: The values of Costa Rica are not shown in figure for clarity (Costa Rica: total occupational injury rate = 8922.5; fatal occupational injury rate = 6.9). The grey line indicates the smooth nonparametric curve using a loess smoother (the loess span 0.75) and the grey shadow indicates the 95% confidence interval of smoothed curve. All data sourced from International Labour Organization Database (ILOSTAT) [18].

Figure 1 represents the fatal and nonfatal occupational injuries based on the statistics in ILOSTAT in each country. The highest total occupational injury rate was 8922.5 per 100,000 workers in Costa Rica and the lowest total occupational injury rate was 26.2 per 100,000 workers in Kyrgyzstan. The country with the highest incidence of fatal occupational accident cases was Mexico (8.2), and that with the lowest incidence was the United Kingdom (0.4).

**Figure 2:**
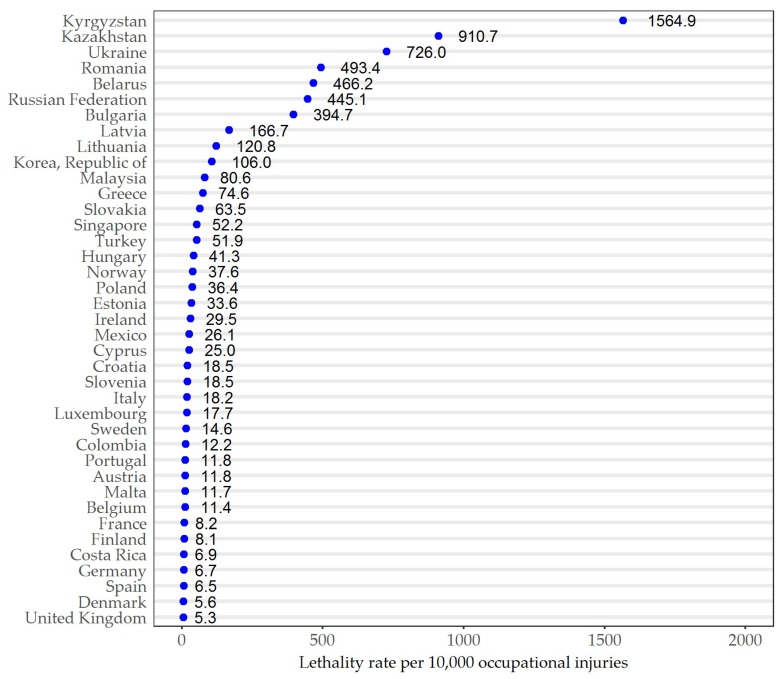
Lethality rate per 10,000 occupational injuries of each country in 2015. Note: All data sourced from International Labour Organization Database (ILOSTAT) [18].

Figure 2 shows the lethality per 10,000 occupational injuries among countries. Kyrgyzstan (1564.9) is the top lethality among the OECD countries, followed by Kazakhstan (910.7) and Ukraine (726.0). The country with lowest lethality was the United Kingdom (5.3).

### 3.2. Factors Associated With Total and Fatal Occupational Injury Rates and Lethality of Occupational Injuries

**Figure 3:**
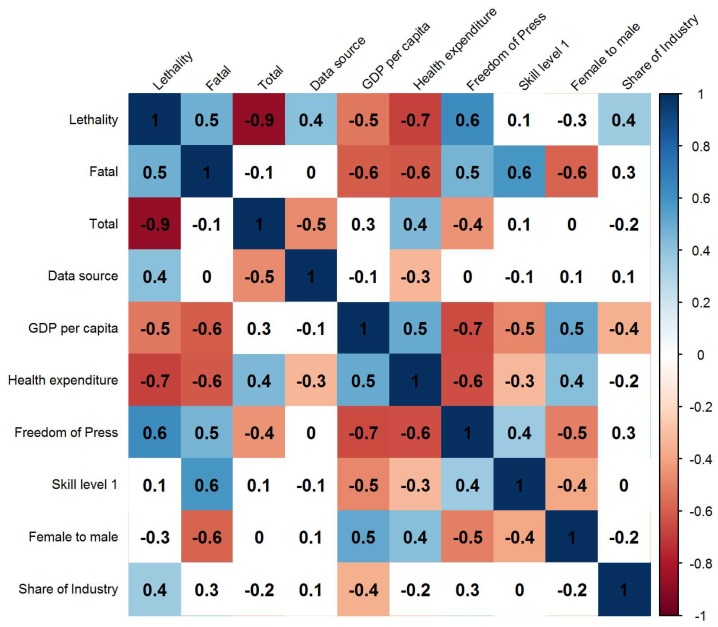
Correlation matrix of summary statistics among 39 countries in 2015. Note: The number indicates the Spearman’s correlation coefficient between two statistics. If there is no colour filled in the box, it indicates that the association between the two variables is not statistically significant (*p*-value < 0.005).

Figure 3 shows the correlation coefficients among summary statistics in 39 countries in 2015. Fatal occupational injury rate was associated with GDP per capita (γ = −0.6, *p*-value ≤ 0.001), CHE as percentage of GDP (γ = −0.6, *p*-value ≤ 0.001), proportion of employment at skill level 1 (γ = 0.6, *p*-value ≤ 0.001) and the ratio of female to male labour force participation rate (γ = −0.6, *p*-value ≤ 0.001). There was no significant association between total occupational injury rate and any of the job-related indicators including proportion of employment at skill level 1 (γ = 0.1, *p*-value = 0.477), ratio of female to male labour force participation rate (γ = 0.0, *p*-value = 0.997) and share of industry in total employment (γ = −0.2, *p*-value = 0.198). Lethality per 10,000 occupational injuries had a significant correlation with data source (γ = 0.4, *p*-value = 0.008), GDP per capita (γ = −0.5, *p*-value ≤ 0.001), CHE as percentage of GDP (γ = −0.7, *p*-value ≤ 0.001) and share of industry in total employment (γ = 0.4, *p*-value = 0.030). Freedom of the press was significantly correlated with fatal (γ = 0.5, *p*-value = 0.003), total occupational injury rate (γ = −0.4, *p*-value = 0.004) and lethality rate of occupational injuries (γ = 0.6, *p*-value ≤ 0.001).

### 3.3. The Association between a Free Press and the Lethality Rate of Occupational Injury

**Figure 4:**
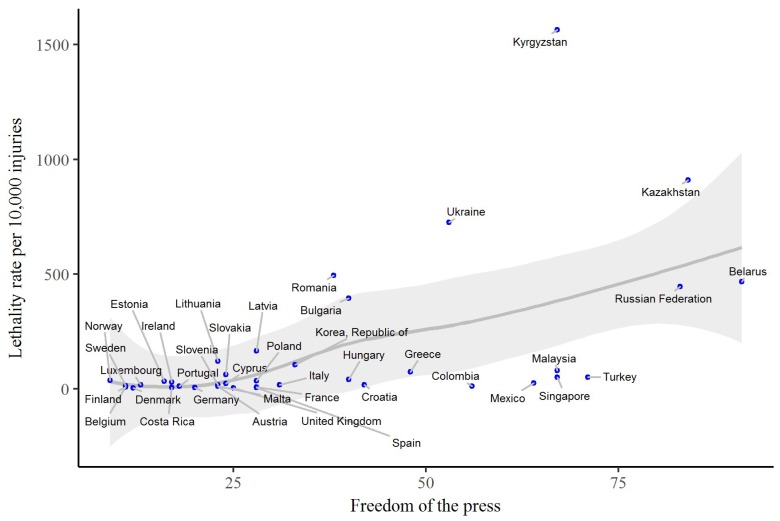
Freedom of the press and lethality rate per 10,000 occupational injuries of 39 countries in 2015. Note: All data sourced from International Labour Organization Database (ILOSTAT) [18] and Freedom House [23]. The grey line indicates the smooth nonparametric curve using a loess smoother (the loess span 0.75) and the grey shadow indicates the 95% confidence interval of smoothed curve.

Figure 4 shows how freedom of the press influences the lethality rate per 10,000 occupational injuries. The score of freedom of the press ranges from 9 (Norway) to 91 (Belarus). There is an increasing trend of the correlation between freedom of the press and the lethality rate of occupational injury.

Table 2 shows the summary of the bootstrap regression results and two approaches of the 95% confidence interval among 39 countries in 2015. After adjusting for national indicators, only freedom of the press was found to be associated with lethality rate per 10,000 occupational injuries (linear coefficient = 7.2; bias = −0.1; 95% percentile CI, 3.1–17.2; 95% BCa CI, 2.0–14.1; 95% CI from normal distribution, 1.7–12.7: not shown in Table 2). We found no significant association between lethality rate and any of the other national indicators.

## 4. Discussion

In this study based on the national indicators from the representative statistical agency, we evaluate whether lethality rates per 10,000 occupational injuries were associated with national or job-related statistics. The national indicators related to lethality rate were GDP per capita, CHE as percentage of GDP (%), freedom of the press and share of industry in total employment (%) in the correlation study. However, after controlling for national statistics, only freedom of the press was associated with lethality of occupational injuries.

In our study, fatal occupational injury rate was not correlated with total occupational injury rate. According to the Heinrich’s law, 0.3% of all accidents cause major injuries, 8.8% cause minor injuries, and 90.9% cause no injuries [30]. This rule represents that the higher the number of nonfatal injuries in a country, the higher the number of fatal injuries, resulting in a relatively constant ratio between minor and fatal accidents. However, in line with a previous study [9], our results of correlation matrix showed that there was no correlation between the two occupational injury indices and no constant ratio between the two indices. Furthermore, in our study, total occupational injury rate among 39 countries based on the statistics in ILOSTAT was not correlated with any of the job-related indictors, while fatal occupational injury rate was correlated with two of the job-related indicators, proportion of employment at skill level 1 and ratio of female to male labour force participation rate. Therefore, nonfatal injuries officially reported by each country might not represent the actual number of incidents and could be distorted due to the problem of under-reporting or administrative procedures. This statistical inaccuracy in nonfatal occupational injuries might be due to lack of work accident records or failure to reveal a hidden occupational injury of workers.

Our results showed that the level of free press was negatively correlated with both lethality rate and fatal occupation injury rate and positively correlated with total occupational injury rate. This might be explained by the fact that workers could not easily reveal hidden nonfatal injuries outside of the workplace in countries where free press is not guaranteed. However, fatal injuries are not as easy to hide within the company or country considering that the impact of the death of a worker would be considerable for their family or country and that deaths in the workplace are normally compensated in most countries [31]. In other words, considering the relationship between press oppression and lethality rate, the actual situation of lethality of occupational injuries in any country is not reflected accurately but is under-reported to government authorities and eventually to the official statistics agency, ILOSTAT. Open and unbiased press acts as a bridge between government and workers to exchange information and lessen this gap of information [16]. The press plays the role of a monitor or ‘watchdog’ for inappropriate behaviours in the workplace [17]. Therefore, repression of the press can make it difficult to reveal the hidden industrial accidents caused by employers’ indifference toward the industrial health of vulnerable employees and by a lack of understanding of the regulations of industrial accidents.

Freedom of the press helps promote the health of workers in many respects. For example, a free press was associated with improved global infectious disease surveillance [32]. In addition, the media monitors a company’s and country’s negative conditions, motivating employer and government authorities to take appropriate actions [33]. If the employer shows any negligence in managing hazardous substances or risk factors for industrial accidents in the workplace, the press can inform the public, making citizens sit up and take notice and lead to correcting the problems. The role of the media is thereby important in managing occupational injuries.

In the correlation matrix of our study, both GDP per capita and CHE as percentage of GDP (%) are associated with the lethality rate of occupational injury, although this relationship is not statistically significant after adjusting for other national indicators. Both figures are indicative of national competitiveness [19,20]. In terms of worker fatalities, a large disparity on unintentional occupational injury mortality exists between high-income countries (3.1 per 100,000 people) and low-income countries (7.0 per 100,000 people) in 2016 [34]. Given this information, it is reasonable to assume that a higher level of national competitiveness will provide more coverage for nonfatal injuries than a lower level of national competitiveness because it will ensure more funds to compensate for industrial injuries. CHE as percentage of GDP (%) was positively associated with total occupational injuries reported in ILOSTAT in our study, despite a negative association between CHE as percentage of GDP and fatal occupational injuries. Countries with high national competitiveness and high expenditure on health also can afford to invest in safety management in the work environment and provide healthcare for workers. Popescu et al. showed a positive correlation between healthcare expenditure and health index (the composite of health status, medical service supply and health indicators related to the labour marker) among the European Union countries [35].

To the best of our knowledge, this is the first study to establish the relationship between freedom of the press and lethality rate per occupational injuries. However, our results should be interpreted within the context of the study’s limitations. First, because the cross-sectional study was conducted in 39 countries in the year 2015, we could not establish a causal relationship between freedom of the press and lethality of occupational injury. In the future, longitudinal analysis is needed to clarify factors related to lethality rate more accurately. Second, it is not enough to establish the association between free press and fatality rate of occupational injury because we used data of national indicators in only one year. Therefore, we applied the same multiple regression method to the data of 2012, 2013 and 2014 (see Supplementary Material: Table S1). Third, the variable of lethality was not normally distributed because of the small number of countries in the study. To compensate for these limitations, we performed the robust method using the Spearman’s rank test and bootstrap technique to establish the relationship between freedom of the press and lethality. Fourth, freedom of the press score may not accurately reflected the country’s actual free press situation. However, this score was validated to some extent by comparison with other reputable organizations’ press freedom scores, for example with Reporters Sans Frontières’ press freedom scores [24]. The freedom of the press score, designed by Freedom House, considers the economic environment, which might be associated with national expenditures (e.g., GDP per capita). However, the economic environment category in the freedom of the press score included most media funding (e.g., the structure of media ownership, transparency and concentration of ownership and the costs of establishing media as well as any impediments to news production and distribution) [23]. Fifth, although the data of representative indicators of global competitiveness or industrial safety published in World Bank, WHO and ILOSTAT were used, it might not reflect the reality of each country, leading to biased results. Sixth, we did not fully adjust for factors related to occupational injury such as the insurance system (public vs. private) or the waiting period of compensation for occupational injuries. The Korea Workers’ Compensation and Welfare Service and Italian National Occupational Health Insurance compensates and reports occupational injuries longer than three days [4,36]. However, the period for compensation for occupational injuries is one day in Denmark, Spain and the Czech Republic [9]. These different waiting periods among countries might result in bias in the reporting of occupational injury rate. Future studies are needed to identity the factors affecting the fatality of occupational injury and the under-reporting of hidden nonfatal occupational injuries.

## 5. Conclusions

This study shows that the lethality rate of occupational injuries reported by each country does not reflect the actual fatality of injury but reflects the under-reporting of nonfatal occupational injuries, which is related to the freedom of the press. Given the tremendous socioeconomic costs of industrial accidents and the loss of work capacity of workers, the government should make efforts to reveal hidden victims of nonfatal occupational injuries and create policies that make it easy for vulnerable workers who do not receive proper social security to inform about occupational injuries to governmental authorities via guaranteeing freedom of the press.

## Figures and Tables

**Table 1:** Variable definitions and summary statistics in the year 2015 among 39 countries.

Variables	Definition	Median (IQR)	Data Source
Lethality	Fatal occupational injuries per 10,000 occupational injuries	29.5 (81.5)	calculated variable from ILOSTAT
Fatal	Fatal occupational injuries per 100,000 workers	2.6 (2.0)	ILOSTAT
Total	Total occupational injuries per 100,000 workers	744.9 (1484.6)	calculated variable from ILOSTAT
Data source	Insurance records = 0, Others = 1	0.6 ^a^ (0.5)	ILOSTAT
GDP per capita	GDP per capita (constant 2011 international $)	27549.6 (15686.8)	World Bank
Health expenditure	Current health expenditure (CHE) as percentage of GDP (%)	7.4 (3.2)	WHO
Freedom of Press	Total score of Freedom of the Press 2016	28.0 (31.5)	Freedom House
Skill level 1	Proportion of employment at Skill level 1	9.6 (4.7)	ILOSTAT
Female to male	Ratio of female to male labour force participation rate	80.3 (10.0)	ILOSTAT
Share of Industry	Share of industry ^b^ in total employment (%)	24.4 (7.6)	ILOSTAT

^a^ indicates the mean and standard deviation of data source; ^b^ industries included mining and quarrying, manufacturing, construction, and public utilities (electricity, gas, and water); interquartile ranges (IQR); International Labour Organization Database (ILOSTAT); gross domestic product (GDP); World Health Organization (WHO).

**Table 2:** The summary of regression coefficients and 95% confidence interval based on the bootstrap ^a^.

Variables	Original ^b^	Bias ^c^	S.E. ^d^	95% Percentile CI	95% BCa CI
Data source	125.6	−6.8	124.5	(−52.6–461.3)	(−94.5–381.0)
GDP per capita×0.001	−7.5	0.2	5.8	(−23.9–0.8)	(−19.3–2.2)
Health expenditure	12.0	2.5	42.4	(−45.5–127.4)	(−52.6–110.7)
Freedom of Press *	7.2	−0.1	3.1	(3.1–17.2)	(2.0–14.1)
Skill level 1	−8.6	1.9	19.9	(−64.5–20.1)	(−49.0–26.5)
Female to male	3.4	0.2	8.5	(−21.8–16.2)	(−15.0–19.0)
Share of Industry	−14.3	3.3	15.6	(−61.2–6.5)	(−44.8–12.7)

^a^ The number of bootstrap replicates = 10,000 among 39 countries in the year 2015; ^b^ indicates the regression coefficient using multiple linear regression; ^c^ indicates the difference between the mean of regression coefficient of 10,000 stored bootstrap samples and the original estimate of regression coefficient of multiple linear regression; ^d^ indicates the bootstrap estimated standard error; * indicates that regression coefficient is statistically significant in both methods on calculating 95% CI (percentile CI, BCa CI); gross domestic product (GDP).

## Supplementary Materials

The following are available online at http://www.mdpi.com/1660-4601/15/12/2856/s1, Table S1: The summary of regression coefficients and 95% confidence interval based on the bootstrap (The number of bootstrap replicates = 10,000) in 2012, 2013, and 2014.
