# Supplementary material for: The Relationship between Free Press and Under-Reporting of Non-Fatal Occupational Injuries with Data from Representative National Indicators, 2015: Focusing on the Lethality Rate of Occupational Injuries among 39 Countries
Source: Int J Environ Res Public Health. 2018 Dec 14;15(12):2856. doi: 10.3390/ijerph15122856 (PMC6313768; doi:10.3390/ijerph15122856)
Supplement: Supplementary file 1 [file ijerph-15-02856-s001.pdf]

Table S1. The summary of regression coefficients and 95% confidence interval based on the bootstrap (The number of bootstrap replicates = 10,000) in 2012, 2013, and 2014.

Variables	Original [a]	Bias [b]	S.E. [c]	95% Percentile CI	95% BCa CI
2012 (*n* = 42)					
Data source	299.2	12.0	272.5	(−1.4–1278.2)	(−25.0–934.8)
GDP per capita × 0.001	−1.0	−1.9	8.9	(−21.6–13.7)	(−24.7–11.1)
Health expenditure	34.9	6.8	71.5	(−36.9–328.6)	(−48.0–213.7)
Freedom of Press *	9.4	−0.3	9.6	(1.4–61.4)	(−4.1–33.2)
Skill level 1	28.1	−1.5	35.0	(−2.6–183.9)	(−16.8–117.0)
Female to male	−1.7	−2.2	13.9	(−70.2–7.8)	(−42.7–10.8)
Share of Industry	−23.1	−1.4	29.5	(−131.1–7.7)	(−93.9–10.7)
2013 (*n* = 43)					
Data source *	313.6	10.0	221.9	(23.4–1076.1)	(−14.2–822.0)
GDP per capita × 0.001	−0.6	−1.6	8.5	(−16.0–17.5)	(−19.4–13.3)
Health expenditure	65.5	7.8	65.0	(−23.3–268.3)	(−29.2–222.0)
Freedom of Press **	13.8	−0.2	6.3	(6.0–40.0)	(3.8–28.7)
Skill level 1	5.2	1.5	22.2	(−29.9–70.1)	(−26.5–60.7)
Female to male	−6.7	−0.9	14.3	(−64.0–9.5)	(−41.8–13.1)
Share of Industry	−13.1	1.4	16.0	(−62.4–9.3)	(−47.5–13.2)
2014 (*n* = 41)					
Data source	343.1	7.0	301.8	(−31.4–1236.9)	(−58.7–1007.8)
GDP per capita × 0.001	1.0	0.1	7.8	(−7.9–26.4)	(−12.1–19.7)
Health expenditure	50.0	4.8	74.4	(−32.7–316.3)	(−44.5–229.9)
Freedom of Press *	8.4	0.4	7.6	(1.3–41.3)	(−1.2–27.9)
Skill level 1	47.2	3.1	46.0	(−2.0–222.2)	(−7.3–160.5)
Female to male	−1.7	−0.3	15.5	(−83.1–11.2)	(−43.8–16.8)
Share of Industry	−2.1	1.3	17.1	(−73.8–15.3)	(−41.4–25.4)

[a] indicates the regression coefficient using multiple linear regression; [b] indicates the difference between the mean of regression coefficient of 10,000 stored bootstrap samples and the original estimate of regression coefficient of multiple linear regression; [c] indicates the bootstrap estimated standard error; * indicates that regression coefficient is statistically significant in one of methods on calculating 95% CI (percentile CI, BCa CI); ** indicates that regression coefficient is statistically significant in both methods on calculating 95% CI (percentile CI, BCa CI); S.E (standard error); CI (confidence interval); BCa (bias-corrected accelerated); gross domestic product (GDP).
